# Animal Models in Neuropsychiatry: Do the Benefits Outweigh the Moral Costs?

**DOI:** 10.1017/S0963180122000147

**Published:** 2022-10

**Authors:** Carrie Figdor

**Affiliations:** Department of Philosophy, Department of Psychological and Brain Sciences, and Iowa Neuroscience Institute, University of Iowa, Iowa City, Iowa 52242, USA

**Keywords:** neuroethics, animal models, neuropsychiatry, research ethics

## Abstract

Animal models have long been used to investigate human mental disorders, including depression, anxiety, and schizophrenia. This practice is usually justified in terms of the benefits (to humans) outweighing the costs (to the animals). The author argues on utility maximization grounds that we should phase out animal models in neuropsychiatric research. The leading theories of how human minds and behavior evolved invoke sociocultural factors whose relation to nonhuman minds, societies, and behavior has not been homologized. Thus, it is not at all clear that we are gaining the epistemic or clinical benefits we want from this animal-based research.

## Introduction

It is a moral truism that you should not treat human beings like lab rats. This article argues that we also should not treat lab rats like lab rats, at least not insofar as neuropsychiatric research is concerned. Such treatment is not justified even if we assume that the intrinsic moral value of human lives always outweighs that of animal lives and that this research was justified by its epistemic (and eventually clinical) consequences in the past. We should phase it out starting now.

I present several independent but mutually supporting arguments for this conclusion. They do not rely on the widely accepted idea that the pain and suffering of animals have moral weight, and that we ought to stop this research to avoid their pain and suffering. For the sake of argument, I will presuppose that no amount of their pain and suffering can outweigh the benefits to humans of this research. As blind chicken arguments show,^1^ when forced to confront our responsibility for making animals suffer for our benefit, we can simply persist in our behavior without moral qualms by eliminating their ability to feel pain. Instead, I argue that the means of continued animal sacrifice do not justify the ends of human benefits because their efficacy toward those ends is no longer clear. We should phase out using animal models because it is in our self-interest to do so. Not incurring additional moral debt is a felicitous side effect. Because moral appeals in themselves only go so far, this approach may raise the probability that the recommended phase-out will actually occur.

## Jointly Sufficient Reasons to Phase Out Neuropsychiatric Research with Animals

In what follows, I present four mutually supporting arguments for the conclusion that neuropsychiatric research using animal models should be phased out. In what follows, “research” refers to research intended to provide knowledge about, and eventual clinical treatments for, human disorders that have a psychological component or comorbidity, that is, mental disorders such as anxiety and depression, Parkinson’s disease, schizophrenia, and addiction.^2^ This may be a wider net than “neuropsychiatric condition,” but the conditions that count as neuropsychiatric are among the paradigm cases of the research I target. Any definition of “mental disorder” that captures these paradigm cases will suffice for my argument. What matters is this: the reason we use animal models in the first place is to obtain epistemic (and eventually clinical) benefits without endangering human lives, and this reason fails in the neuropsychiatric case. I set aside the possibility of arguing in similar fashion for phasing out animal models in biomedical research for disorders that do not have psychological components. Almost any disorder will have psychological and social consequences, but that is another issue.

### The Consent Problem as Background

No discussion of the use of animals in neuropsychiatric research can avoid the fundamental starting point that the benefits go to the humans while the animals bear all the costs, usually including death in order to examine their brains. This is to say the animals are forced into extreme altruistic behavior, defined in general as behavior that benefits others at the personal cost of the behaving individual.^3^ This moral problem is avoided with humans via the construct of informed consent. A human can agree to be used as an experimental subject without any expected benefit for themselves. Such use is morally permissible if appropriate consent procedures are followed. Animals are not asked if they consent—they are involuntary “martyrs for medicine.”^4^ Even if human consent is ethically required for using animal-derived medical treatments on someone,^5^ the animals are not considered at all.

Of course, it is a presupposition of animal research that the animals are not capable of consent, therefore the very idea of their providing informed consent cannot even arise.^6^ Whether this is true is questionable: at the very least, animals do not go willingly to their deaths. The possibility of consent includes the possibility of always refusing. With humans, a similar inability to consent does not entail the permissibility of indiscriminate treatment. The responsibility for informed consent is transferred to a guardian. But if there is no one to consent on behalf of a human subject, the moral default is that the human cannot be a research subject. Thus, the facts that lab animals cannot offer consent in the usual way, are not given the opportunity to refuse in their own ways, and are not associated with humans who can consent on their behalf (e.g., via ownership) do not make their use as research subjects morally permissible.

Ultimately, the moral permissibility that obviates consent in the case of nonhumans rests on the conviction that human lives are morally more valuable than those of animals. The moral status of humans simply outweighs that of any nonhuman. Whether this traditional view is morally justified is dubious.^7^ But even if we assume the traditional moral status difference, it is also widely accepted that animals have some claim to minimally moral treatment. This is due to an ability to feel pain combined with a moral norm that pain should not be inflicted unnecessarily. But when is inflicting pain necessary? For humans, undergoing surgery causes pain (usually during recovery), which is a known side effect of a necessary means to regaining health. The same goes for human subjects research where pain is likely or inevitable: humans may be voluntary altruists. But with research animals, the pain endured by the animal is involuntary. We are back to the issue of consent. It seems lab animals are stuck providing humans with benefits because humans have higher moral status.

Because the conviction that humans have higher moral worth than nonhuman animals is so ingrained, the consent problem is unlikely to persuade many that animal-based research for neuropsychiatric conditions should be phased out. We do not need their consent, even if they or a human proxy could give it, because our high intrinsic moral worthiness suffices to justify the animals’ sacrifice for our benefit given their low moral worthiness.^8^ Is it true that no amount of animal moral worth can ever amount to the moral worth of humans? Do the values of the lives of vast numbers of animals used in research *never* amount to enough to outweigh the moral value of a human? Perhaps making this comparison itself is so offensive to our sense of human moral value that we cannot even consider it.

I propose to simply grant these moral convictions for the sake of argument. Trying to end or curtail research using animals by overturning these convictions is almost certainly a lost cause. Even if we asked for their consent, their refusals would fall on deaf ears. In a moral version of Zeno’s paradoxes, I will suppose that no amount of adding up animal moral statuses can ever quite reach the moral status of a human. What will be more persuasive is to argue that humans are not in fact benefiting as much as we may think we do from animal-based research. Then the question becomes tractable. If humans are not getting the benefits we want from this research, then the reason for pursuing it falls apart. We are better off seeking alternative means. If we also avoid incurring further moral debt, so much the better. If the benefits we gain from animal-based research are marginal at best, we may as well change our research habits to something more consonant with our moral values—even if we continue to value human lives more than animal lives.

## A Three-Stage Argument Against Animal Models in Neuropsychiatry

In this section, I present three further overlapping considerations that jointly lead to the conclusion that we should phase out animal models in neuropsychiatric research. The epistemic benefits we hope to get from them are not clearly justified, so it is in our self-interest to seek this information by other means.

### The Sociocultural Environment Problem: Neuropsychiatric Disorders Depend on Specific Human Sociocultural Factors, Even Given Neural and Behavioral Homologies Across Species

Our leading current theories of human cognitive (or broadly psychological) evolution posit that our minds are the result of a complex process of gene-culture coevolution, in which sociocultural factors play an essential and primary role.^9^ For example, Cecilia Heyes’ theory of “cognitive gadgets,” or “distinctively human cognitive mechanisms,” holds that such abilities as mind-reading and language have the forms they do because of how they are shaped in evolution by human methods of social and cultural information transmission.

Although sociocultural evolution theorists may also make claims about the uniqueness of these advanced abilities, it is not critical in this context that they be considered uniquely human. If they were, then using animal models for disorders of uniquely human psychological abilities would be a nonstarter, even if human brain function is an important factor in their etiology. Rather, I will assume that there are homologies in brains and behavior relevant to neuropsychiatric conditions. Even so, these theories imply that brain similarities are less relevant to specifically human mental disorders than sociocultural factors, given the importance of the latter in phylogeny as well as ontogeny. A brain that is not primed for human sociocultural contexts will not be a brain that can be disordered in relation to those contexts. It can be disordered in similar, perhaps homologous, ways, but what is likely very critical in human neuropsychiatric disorders may be the sociocultural factors specific to us as an evolutionary matter, as well as in development.

### The Ignorance Problem: We Are Not Justified in Assuming Experimentally Induced Behaviors in Lab Animals Are Relevantly Similar to the Human Behaviors Associated with Human Neuropsychiatric Disorders

The Sociocultural Environment problem points to the need for greater attention to the social and behavioral aspects of neuropsychiatric disorders. But even if lab experiments could in principle capture some relevant similarities with human behavior, we are likely designing experiments that do not in fact capture those similarities. We may induce animal behavior that we interpret as depression-like or optimism-like, but what makes it “like” is not theoretically grounded in established or even hypothesized homologies of socioculturally relevant human behaviors and animal behaviors. Stress hormones are homologous and levels of stress hormone can be measured across species. But depression-like behaviors are not homologous just because we use the same neuropsychiatric labels across species. In part, our ignorance is due to the fact that research funding that gets funneled to animal-based lab research is funding not available for investigating sociobehavioral homologies relevant to neuropsychiatric disorder—or behavioral homologies in general. Whatever the reason, however, it would be pure luck if the animal behaviors we now induce in the lab are in fact homologous to the human ones they mimic.^10^ For we do not know what aspects of the human sociocultural conditions might be replicated in experimental paradigms and so whether our experiments with lab animals are in fact informative in the way they are intended. It follows that lab animals are likely undergoing experimental procedures that do not provide the epistemic benefit we are sacrificing them for.

As a side note, the so-called translation problem of transferring the knowledge we gain from animal-based research to human clinical contexts has already been raised for nonpsychiatric biomedical research.^11^ Such concerns easily extend to the knowledge gained in neuropsychiatric research and its transfer to human mental health contexts. Difficulty in translating animal-based research to humans is a general problem, but neuropsychiatric research is very likely to anchor the “unhelpful” end of this continuum.

### The Granularity Problem: We May Have Already Passed the Point Where Animal Models Can Provide Epistemic Benefits Relevant to Neuropsychiatric Disorders

The calculus of justifying animal research is dynamic, as is any cost–benefit analysis. In a past state of ignorance about neurobiology, the moral dangers of human subjects research may once have justified using animals instead, given the moral status differential noted above. This general point will certainly apply to neuropsychiatric research in particular. There is no doubt that knowledge about neural structure and function, and its biological context from genes to behavior, has increased tremendously in recent decades. We have gained many epistemic benefits from this research, some of which have accrued to neuropsychiatry and resulted in novel pharmacological treatments. But while other animals can tell us a great deal about humans with respect to what we have in common, at some point human subjects are essential for progress. The epistemic benefits of animal research in any biomedical domain diminish over time, and human subjects are required for the fine-grained, species-specific information that is our ultimate goal. There may still be plenty to learn about chickens or mice, but its relevance to humans inevitably becomes increasingly marginal at best.

Because of the phylogenetic and developmental links between human sociocultural factors, human brains, and human cognition and behavior, this point of no epistemic returns is likely to be reached far sooner in neuropsychiatric research than in biomedical research that aims at physical disorder. When it comes to neuropsychiatric research, social and behavioral factors have long been sidelined in favor of the neurophysiological factors that can be manipulated in the lab using animal subjects.^12^ But the cost–benefit calculus today is not what it was a few decades ago. It is likely that this research has already passed the point where it contributes to the goal of learning about, and ultimately treating, human neuropsychiatric disorder. Further fine-grained knowledge of neurobiological mechanisms will do little to address the yawning gaps in our knowledge of the relevant sociocultural factors behind human mental disorder, the relevant homologies between human behavior and induced lab animal behavior, and the species-specific aspects of human neuropsychiatric conditions. We would be better off seeking the knowledge we want with other types of research.

Bringing more human subjects into research pools is one alternative, despite all the moral concerns that must be addressed. Using computer simulations with human avatars is another. What is critical is to recognize that what maximized utility in the past does not automatically maximize utility in the future. Acknowledging that our epistemic self-interest may no longer best be served using animal models is the first step. It promises to put us on the road to phasing out animal research and thereby avoiding its moral costs.

## Conclusion

I have argued that the continued use of animal models for neuropsychiatric research should be phased out. It is not clear that their sacrifices lead to the benefits for humans that allegedly justify the use of animal models in the first place. A summary of my argument is as follows:

P1. We force animals into involuntary altruism for our epistemic benefit, based on a conviction that our lives are more valuable.

P2. Ceteris paribus, if we can get the desired epistemic benefits by means that do not require involuntary altruism, we should.

P3. Human neuropsychiatric disorders depend on human psychology, which is shaped by human sociocultural factors in phylogeny and ontogeny. At best animal proxies only capture similarities (homologies or analogies), be that brains or behavior.

P4. We are relatively ignorant of the relations between artificially induced lab animal behaviors and human behaviors shaped by human sociocultural factors. Our assumption of relevant similarity, in particular homology, is not clearly justified.

P5. So the animals’ involuntary altruism to date may be a waste due to poor experimental design relative to our ultimate epistemic goals.

P6. But even if these experiments have provided valuable knowledge about neurophysiology and neural structure, we are likely near or have passed the point where animal models can provide the fine-grained knowledge we want regarding human neuropsychiatric disorder, based as it is in sociocultural factors.

Conclusion: We should phase out animal models in favor of better research protocols.

A morally beneficial side effect of this shift would be that we would no longer be forcing animals into involuntary altruism. Perhaps it is sad that no longer engaging in morally questionable treatment of animals is, in effect, a moral cherry atop a utility-maximizing cake. But we would not be engaging in morally questionable means to get out of the problem, as with the blind chickens. We would be changing our behavior in a way that yields a morally beneficial byproduct. That is enough of a win for me.

## Notes

1.

Thompson P. The opposite of enhancement: Nanotechnology and the blind chicken problem. Neuroethics 2008;2:305–16.

2.

Willner P. The validity of animal models of depression. Psychopharmacology 1984;83:1–16 642969210.1007/BF00427414; Castner S , Goldman-Rakic P , Williams G. Animal models of working memory: Insights for targeting cognitive dysfunction in schizophrenia. Psychopharmacology 2003;174:111–2510.1007/s00213-003-1710-915205882; Warnick J , Huang C , Acevedo E , Sufka K. Modeling the anxiety-depression continuum in chicks. Journal of Psychopharmacology 2009;23:143–561851545210.1177/0269881108089805; Fernando A , Robbins T. Animal models of neuropsychiatric disorders. Annual Review of Clinical Psychology 2011;7:39–61 10.1146/annurev-clinpsy-032210-10445421219191; Sufka K , Weldon M , Allen C. The case for animal emotions: Modeling neuropsychiatric disorders. In Bickle J , ed. The Oxford Handbook of Philosophy and Neuroscience. Oxford: Oxford University Press; 2009 ; Decourt M , Jimenez-Urbieta H , Benoit-Marand M , Fernagut PO . Neuropsychiatric and cognitive deficits in Parkinson’s disease and their modeling in rodents. Biomedicines 2021;9:684 3420438010.3390/biomedicines9060684PMC8234051. doi:10.3390/biomedicines9060684; Nelson N. *Model Behavior: Animal Experiments, Complexity and the Genetics of Psychiatric Disorders.* Chicago: Chicago University Press; 2018.

3.

Kerr B , Godfrey-Smith P , Feldman, M. What is altruism? Trends in Ecology and Evolution 2004;19(3):135–401670124410.1016/j.tree.2003.10.004.

4.

Ratnoff M , Smith J. Human laboratory animals: Martyrs for medicine. Fordham Law Review 1968;36(4):673–94.

5. Rodgers D. Why we should stop using animal-derived products on patients without their consent. *Journal of Medical Ethics* 2021 doi:10.1136/medethics-2021-107371.

6.

Martin A. Animal research that respects animal rights: Extending research requirements for research with humans to animals. Cambridge Quarterly of Healthcare Ethics 2022;31(1):59–72 3504945510.1017/S0963180121000499.

7.

Figdor C. The psychological speciesism of humanism. Philosophical Studies 2021;178:1545–69.

8. Angela Martin (see note 6) argues that animal research should be more aligned with the constraints on human subjects research if they have the same moral worth as humans. This is too big an “if” for many. And while Martin is correct in principle that stopping the research might mean the animals themselves would not get any benefits, in practice it is unlikely that much benefit would accrue to the animal subjects (or their descendants) and questionable whether it could ever outweigh the sacrifices made to get it. However, Martin’s paper is concerned with animal research in general, in particular nonharmful and low-risk research. In such cases her arguments are stronger.

9.

Boyd R , Richerson P. Culture and the Evolutionary Process. Chicago: Chicago University Press; 1985 ; Sterelny K. Thought in a Hostile World: The Evolution of Human Cognition. Oxford: Oxford University Press; 2003 ; Heyes C. *Cognitive Gadgets.* Oxford: Oxford University Press; 2018.

10. A major research initiative (https://sites.google.com/view/socialroots/the-project) has been launched to address some of these concerns.

11.

Sullivan J. The multiplicity of experimental protocols: A challenge to reductionist and non-reductionist models of the unity of neuroscience. Synthese 2009;167:511–39; Bolker J. Animal models in translational research: Rosetta stone or stumbling block? Bioessays 2017;39:170008910.1002/bies.20170008929052843; Piotrowska M. From humanized mice to human disease: Guiding extrapolation from model to target. Biology and Philosophy 2013;28:439–55.

12.

Ankeny R , Leonelli S. Model Organisms. Cambridge: Cambridge University Press; 2020 ; see note 2, Nelson 2018.

